# Mandatory vaccination: suited to enhance vaccination coverage in Europe?

**DOI:** 10.2807/1560-7917.ES.2019.24.26.1900376

**Published:** 2019-06-27

**Authors:** Heidemarie Holzmann, Ursula Wiedermann

**Affiliations:** 1 Center for Virology, Medical University of Vienna, Austria; 2 Institute of Specific Prophylaxis and Tropical Medicine, Medical University of Vienna, Austria

**Keywords:** mandatory vaccination, vaccines, immunisation, vaccination, Europe

Vaccines are one of the most successful medical measures that save millions of human lives every year. With the implementation of routine immunisation programs, high and maintained vaccination coverages for many vaccine-preventable diseases—such as those against poliomyelitis or diphtheria—have been reached in most European countries and many others [1,2]. Although vaccine acceptance is often high within the general population, even in countries with high vaccination coverage a significant number of children and adults are not sufficiently vaccinated because of missed opportunities or various concerns and misperceptions. The reasons for this ‘vaccine hesitancy’ are multifactorial, complex and vary across vaccines, time and countries/regions, and are influenced by factors such as complacency (not perceiving disease as high risk and vaccination as necessary), convenience and constraints (practical barriers), and confidence (lack of trust in safety and effectiveness) [3-6]. As a result, vaccination coverages against highly contagious pathogens such as measles virus are not sufficient to prevent outbreaks and infectious disease spread in many countries today.

Despite the World Health Organization (WHO)’s goal to eliminate measles [7,8], a constant increase in measles cases has occurred in recent years. In 2018, more than 82,500 people in 47 of the 53 countries in the WHO European Region were infected with measles, leading to 72 deaths. These numbers were the highest in a decade. They were three times higher than in 2017 and 15 times higher than in 2016, when numbers were at a record low [9-11]. In 2019, the situation seems to be even worse [12,13], indicating that current plans of action in the affected areas are insufficient to stop measles circulation. This is evidenced by the fact that the estimated coverage with the second dose of a measles-containing vaccine is far below the necessary 95% to achieve herd/population immunity in several European countries [13]. In order to maintain or improve the population immunity acquired by vaccination, several countries are currently revisiting their strategies and discussing changes in vaccination policies, with a focus on either educating the population and giving individuals freedom of choice or implementing mandatory vaccination to ensure high coverage rates [14].

With increasing calls to introduce mandatory vaccination programs, intense debates on their effectiveness have also started in several European countries. There are concerns that mandatory vaccination may lead to opposing attitudes and even less vaccine uptake, particularly in those with existing critical attitudes towards vaccines [15]; nonetheless, other studies have disproved that implementation of compulsory vaccination led to opposing attitudes and/or had negative effects [14]. However, it is indisputable that with any changes in vaccination policies, intensified information strategies are necessary to improve trust, rectify perceived risks and improve access and affordability of vaccines [3,15]. Moreover, it is important to note that mandatory vaccination can follow different routes depending on a country’s specific social and cultural backgrounds, as well as epidemiological situations. Consideration of these factors can lead to implementing temporary or permanent vaccine mandates for certain vaccines (such as measles/measles-mumps-rubella (MMR) partial compulsory vaccination [15]), for all vaccines included in a national vaccination program [14]] or for selected target groups, such as infants and children before entrance in educational settings or certain occupational groups, such as healthcare workers (HCW) [16].

For example, in France three mandatory vaccines (against diphtheria, tetanus and poliomyelitis (DTP)) co-existed with eight recommended vaccines (against MMR, pertussis, *Streptococcus pneumoniae*, hepatitis B (HepB), *Neisseria meningiditis* serogroup C (MenC) and *Haemophilus influenza* (Hib)) for routine childhood immunisation up until 2017. However, misperceptions in the population, i.e. that non-mandatory vaccines are less valuable, optional or not as safe and effective as the mandatory ones, resulted in insufficient and stagnating vaccine coverages of the recommended vaccines. This growing vaccine hesitancy, as well as large outbreaks and deaths from measles, led to a change in French policy to extend the mandates to all 11 childhood vaccines [17].

Italy has had a similar situation, where four mandatory vaccines were in place already before 2017 (against poliomyelitis, tetanus, diphtheria and HepB). The coverage for vaccination against measles, mumps and rubella dropped country-wide from 90% to 87% between 2000–16 [18,19]. This, together with large measles outbreaks, led the government to extend the existing vaccine mandates to 10 mandatory vaccines (hexavalent vaccine against DTPert (pertussis)-poliomyelitis-Hib-HepB, as well as MMR and Varicella (V) vaccine) in 2017, whereas vaccination against Men C, *S. pneumoniae* and rotavirus remained recommended vaccines.

The current issue of *Eurosurveillance* presents articles from France and Italy on approaches and experiences after the extension of mandatory vaccination [19,20]. While an article in last week’s issue of *Eurosurveillance* by Mathieu et al describes the population’s general attitude towards mandatory vaccination shortly before implementation of extended vaccination mandates in France [21], the rapid communication by Lévy-Bruhl et al. in this issue evaluates the effects of mandatory vaccination on vaccine coverage 2 years after its implementation [20]. D’Ancona et al., also in this issue, depict challenges in Italy in the year following the introduction of the new mandate and how these are being addressed [19].

Mathieu et al. performed a cross-sectional survey among 3,222 individuals in France, at the time of implementation of the new law, to assess attitudes towards the new vaccination policy and factors associated with a favourable opinion [21]. More than two thirds of survey participants agreed with the extension of the vaccine mandates, considered it as a necessary step and assigned a higher value to these vaccines. However, around 57% deemed the law as authoritarian. The article by Lévy-Bruhl et al. illustrates the impact of the extended mandates on the vaccination coverages of children born in 2018, as well as for vaccines not concerned with the law, such as the HPV vaccine [20]. The legislation stipulates that non-vaccinated children cannot attend any kind of collective institutions, such as nurseries, kindergartens or schools, and no reasons for refusal other than medical exemptions are possible. Regardless of initial debates and concerns regarding whether this compulsory mode of action would foster anti-vaccination stances, already 1 year after implementation the vaccination coverages increased for the mandatory vaccines. The sharp increase in Men C vaccination coverage (36.4%) resulted in a notable decrease of cases of invasive meningococcal C disease. Of particular importance is the finding that vaccination coverages also increased for non-mandatory vaccines, such as the HPV vaccine, as well as in older children not covered by the mandates. The authors conclude that this reflects the commitment and efforts of the government to conduct intensive information campaigns along with the new law. In particular, establishing a governmental website dedicated to vaccination helped to provide answers to common questions on vaccines and vaccination, thereby building trust and improving confidence in safe and effective vaccines [20].

In Italy, the extended mandatory vaccination program has been implemented following large measles outbreaks in 2017. Ten vaccines are now compulsory for admission to daycare, kindergarten and schools along with financial sanctions for parents/guardians of children between 6–16 years of age who have not followed the new law. Within 24 months of extended mandatory vaccination, the coverage rates for the mandated vaccines increased between 3–7%. With regard to measles [19], the required coverage rate of 95% has been nearly reached within the past 2 years. Despite this measurable improvement in coverage rates, debates are still ongoing in certain areas of the country because of perceived constraints of individual freedoms and an authoritarian modus operandi in public health aspects [19]. With the recent change of the government, the Italian parliament is now discussing a new legislative proposal, which might reduce mandatory vaccination to measles vaccination only.

These experiences from France and Italy show that mandatory vaccination may even face challenges in countries with a long-standing history of compulsory preventive measures and highlight the need for accompanying activities such as targeted communication and support, e.g. introducing electronic vaccination registries with reminder functions. In view of the high incidence of measles cases in Germany and Austria in recent years, both countries with vaccination programs that do not have vaccine mandates, discussions on the pros and cons of mandatory vaccination are ongoing among experts and in public media. Questions have arisen whether compulsory vaccination (partial or general) might lead to resistance related to people’s fear of unwarranted adverse effects, with a further decline in vaccination coverage, rather than helping to increase coverage rates [15,22].

Alternative strategies could focus on mandatory vaccination for children at entrance into collective/public institutions such as childcare centres, kindergartens, schools, etc., but with the possibility to opt-out, leaving the autonomous decision intact [23]. Some countries, such as Finland, achieved high vaccination coverages for recommended vaccines without mandatory vaccination but with the help of comprehensive electronic vaccination registries and recall systems, along with easy access to vaccinations, e.g. physicians proactively addressing patients and applying motivational interviewing skills, vaccination by occupational physicians at work places or by nurses or pharmacists. Focus on mandatory vaccination was only on HCW, which, however, falls under the responsibility of the respective employer rather than the public health authorities [24].

Studies and surveys have consistently shown that the key persons for vaccine uptake, transmission of information and clarifications are physicians and HCW, who act as trusted role models whose advice is followed by parents/guardians and patients. Therefore, profound education of medical students in vaccinology and further training of physicians of all disciplines—as well as of other HCW—is a high priority to improve their knowledge and strengthen their own positive attitudes towards vaccines [16]. Recent outbreaks of vaccine-preventable diseases (such as measles) have on many occasions involved HCW, and infected HCW constitute a particular risk for their patients, both in hospital and ambulatory settings [16]. Thus, medical and ethical obligation of self-protection and prevention of transmission to others, in particular vulnerable population groups, might justify standard guidelines for necessary vaccines according to risk exposure and implementation of mandatory vaccination of HCW, along with the necessary infrastructure and logistics to facilitate compliance with such regulations. Recently published reviews have shown that acceptance of vaccines even increased after the introduction of compulsory vaccinations among HCW [14,16].

In conclusion, mandatory vaccination cannot be implemented under a uniform procedure and might not be a solution for all countries because of different target groups with differing ages and social, cultural, psychological and educational backgrounds within the populations. During continuous large outbreaks it might be necessary, however, to temporarily control disease spread through vaccine mandates for children and highly exposed groups in educational and public health facilities in order close vaccination gaps and stop transmission. As vaccination gaps in adolescents and young adults exist in several European countries, the introduction of mandates for the infant/childhood immunisation programs might, however, not be suited to instantly close the immunity gaps in these age groups [25-27]. Therefore, supplemental immunisation activities are urgently needed to increase the coverages in these age groups. Importantly, these strategies need to be accompanied by advocacy, trust-supporting communication or electronic vaccination registries/recall facilities. With regards to HCW, there is a broad consensus among European experts that mandatory targeted vaccination would minimise risk of infection and transmission of vaccine-preventable diseases within the healthcare setting [14].
